# Questions asked and answered in pilot and feasibility randomized controlled trials

**DOI:** 10.1186/1471-2288-11-117

**Published:** 2011-08-16

**Authors:** Milensu Shanyinde, Ruth M Pickering, Mark Weatherall

**Affiliations:** 1 Primary Care and Population Sciences, University of Southampton, Southampton General Hospital, Tremona Road, Southampton, UK; 2 School of Medicine and Health Sciences, University of Otago Wellington, Wellington, New Zealand

## Abstract

**Background:**

In the last decade several authors have reviewed the features of pilot and feasibility studies and advised on the issues that should be addressed within them. We extend this literature by examining published pilot/feasibility trials that incorporate random allocation, examining their stated objectives, results presented and conclusions drawn, and comparing drug and non-drug trials.

**Methods:**

A search of EMBASE and MEDLINE databases for 2000 to 2009 revealed 3652 papers that met our search criteria. A random sample of 50 was selected for detailed review.

**Results:**

Most of the papers focused on efficacy: those reporting drug trials additionally addressed safety/toxicity; while those reporting non-drug trials additionally addressed methodological issues. In only 56% (95% confidence intervals 41% to 70%) were methodological issues discussed in substantial depth, 18% (95% confidence interval 9% to 30%) discussed future trials and only 12% (95% confidence interval 5% to 24%) of authors were actually conducting one.

**Conclusions:**

Despite recent advice on topics that can appropriately be described as pilot or feasibility studies the large majority of recently published papers where authors have described their trial as a pilot or addressing feasibility do not primarily address methodological issues preparatory to planning a subsequent study, and this is particularly so for papers reporting drug trials. Many journals remain willing to accept the pilot/feasibility designation for a trial, possibly as an indication of inconclusive results or lack of adequate sample size.

## Background

In the last decade a number of authors have reviewed the justification for describing a trial as a pilot or feasibility study in terms of its content and the questions it addresses [1-5]. Lancaster et al [2] list as legitimate objectives of a pilot study: sample size calculation; providing a dummy run of trial procedures/the protocol; testing data collection forms or questionnaires; testing how randomization procedures work; determining recruitment and consent rates; examining the acceptability of the intervention; and selection of the most appropriate primary outcome measure. Thabane et al [5] categorize reasons to conduct a pilot study into four groups: assessing the feasibility of processes that are key to the success of the main study; assessing time and resource problems; potential human and data management problems; and scientific issues including the assessment of treatment safety, dose, response effects and variance of the effect. They also include a checklist of items to include in reports of pilot studies. Arain et al [4] recommend the NIHR Evaluation, Trials and Studies Coordinating Centre definitions [6] which describe a pilot study as a miniature version of a main study run to test whether components of the main study work together, while feasibility studies are pieces of research done before a main study to answer the question "Can this study be done?". According to these definitions both pilot and feasibility studies play a preliminary role in the design stage of a subsequent larger trial, and do not themselves address efficacy.

Arain et al [4] comment that researchers applying for funding for trials inadequately powered to address clinically meaningful hypotheses may adopt the designation of a pilot study in the hope of a more favourable review. When studies are prepared for publication authors may similarly believe that labeling a small trial as a pilot increases its chance of acceptance. Although Arain et al found that the editors of five high ranking medical journals did not encourage publication of pilot studies because of their perceived lack of rigour, it is possible that other journals are more accommodating. In an editorial in the journal Circulation, Loscalzo [7] proposed a binary classification: trials designated *a priori *as pilots, and those redefined *a posteriori*. During a five year period 41 pilot trials were published in the journal. Many had been designated as pilots at the request of the editorial office to alert readers to uncertainty in the generalizability of their results and their preliminary and exploratory nature. Such a policy is likely to result in trials primarily addressing efficacy being described as pilots, contrary to the NIHR and other recent definitions.

In comparison to the well established pathways of development for pharmaceuticals, prior to 2000 there was little specific guidance on the development of procedures involved in trials of non-pharmacological interventions, possibly because of their complex and heterogeneous nature. The MRC guidelines for the evaluation of complex interventions published in 2000 [8] and revised in 2009 [9] emphasize the importance of testing procedures before planning an evaluation, and also the circular nature of development, feasibility and piloting, evaluation, implementation, and further development. There is no specific guidance for non-pharmacological interventions that do not meet the MRC definition of complexity. In this paper we review a random sample of 50 papers reporting randomized controlled trials (RCTs) published in journals covered by the MEDLINE and EMBASE databases where authors described their trial as a 'pilot' or addressing 'feasibility' in the title. We review papers published between 2000 and 2009, the ten years following the publication of the first MRC guidelines on complex interventions. We examine stated objectives, results and conclusions drawn, and in particular whether these relate to methodological issues, efficacy or safety/toxicity: comparisons are drawn between papers reporting drug and non-drug trials.

## Methods

We searched the EMBASE and MEDLINE databases on 29^th ^July 2010 to identify papers reporting parallel group trials with one or both of the words 'Pilot' and 'Feasibility' in the title. To be included, papers had to be published between 2000 and 2009, written in English, studying humans and indexed as an RCT. Using computer generated random numbers we selected 50 of those identified for full review [10-59]. The sample size was chosen taking into account resources available and the detailed review required, it allows percentages between 10% and 17.5% to be estimated with a 95% confidence interval (CI) of ± 10%. The search was repeated on the 21^st ^February 2011 to gain a more complete estimate of the number of relevant papers in 2009: two papers were selected from those identified on this date to replace papers found to be ineligible at a late stage.

A form was designed to document characteristics of the selected papers. It was tested by all three authors on three randomly selected papers which were not part of the main review sample. Minor modifications were made after the first two papers of the main sample had been reviewed. We defined drug trials as those involving the administration of a discrete chemical entity, substance, or biological agent by mouth or other route, for example by injection. Questions on the form related to: any blinding and in particular double blinding; the numbers of active and placebo/control arms; whether multiple centres were involved (sometimes deduced from the number of institutional review boards mentioned); the target and actual sample sizes; and any justification given for the target sample size.

Research objectives stated in the Abstract and Introduction sections were coded as relating to methodological issues, efficacy, or safety/toxicity, as were statements summarizing results and conclusions chosen for inclusion in the Abstract. Objectives had to be explicitly stated, it was not enough for the reviewer to deduce what the objectives might have been from results presented, or conclusions drawn. Efficacy conclusions in the Abstract were rated as indicating that the experimental intervention had not been shown to have benefit or had no benefit, that it showed promise, or that it showed actual benefit. The accuracy of conclusions drawn by the authors was not verified. An example of an efficacy conclusion in the Abstract rated as indicating the intervention had not been shown to have benefit or had no benefit is

"*With the numbers studied, we failed to find a significant difference between the two groups; thus we have no evidence of a benefit from botulinum toxin injection in the treatment of chronic tennis elbow*" [34];

one rated as indicating the intervention to have promise is

"*Therefore, the colonic colplasty seems to be an attractive pouch design because of its feasibility, simplicity, and effectiveness." *[31];

and one rated as indicating the intervention to have actual benefit is

"*Sleep educational programs for secondary students are recommended to improve information about sleep*." [23].

Numerical results presented in the Results sections were classified as relating to methodological issues, efficacy, or safety/toxicity. We looked for counts of trial participants experiencing methodological problems or side effects, or numerical summaries of statistical findings: text statements that a procedure was feasible for example were not enough to qualify as a result. Methodological results were recorded separately for: recruitment, retention, compliance/adherence to intervention, blinding procedures, acceptability of the intervention to participants, other aspects of the intervention, outcome assessment, logistics of the randomization procedure, acceptability of trial procedures, or the logistics of multi-centre procedures. Selection of these topics was in part based on the issues that Lancaster et al [2] list as constituting pilot studies. We checked the Methods sections to see whether methodological results were reported there, sometimes they formed part of a CONSORT flowchart [60] for example. Depth of coverage was coded as none, brief, detailed or tabulated/graphical presentation. Significance tests and CIs presented for efficacy outcomes were examined to see whether they indicated between or within group significant differences, but no attempt was made to judge which findings were the primary evaluation of efficacy.

In the Discussion sections we again rated coverage of methodological issues, efficacy, and safety/toxicity as none, minimal, substantial, or the major focus of the section. An example of discussion concerning methodological issues rated as minimal was

"*The results are promising but not conclusive because of the low numbers of patients studied, and we recommend that a sufficiently powered study should be performed." *[29];

and the following example was rated as substantial

"*In order to show an assumed clinically relevant difference of 2 kg, with an 80% power and a type-I error of 5%, 300 patients would be needed (150 in each treatment group). If a subsequent study were to be planned, it would be advisable to use the mean change in grip strength as a primary variable because the variability for this parameter was lowest in the present study and it came close to identifying a significant difference between groups (p = 0.196). In addition, grip strength is a quantifiable measurement of effect, unlike the more subjective measurement of pain*." [34].

The NIHR definitions indicate that pilot and feasibility studies should be preliminary research prior to a main study: we were therefore interested in whether authors stated they were conducting a further trial (or were scheduled to start one in the near future). If this was mentioned it was usually in the Discussion section. Since the above two quotes were the only mention of future trials in each paper we did not consider either set of authors to be actually conducting a future trial. Comments in the Discussion concerning lack of power or small sample size were noted.

Finally we recorded whether Conclusions sections contained statements concerning methodological issues, efficacy, or safety/toxicity. The Conclusion section could be a specifically labelled section, a paragraph of the Discussion clearly listing conclusions, or presented as a box: where there was no such section missing was coded not the absence of a relevant conclusion.

The 50 papers were assessed by MS and difficulties arising were discussed with RMP and MW. Blyth-Still-Casella 95% CIs for single percentages and exact CIs for Rate Ratios (RR) were obtained in StatXact [61]. Ordinal ratings were compared between groups in Mann-Whitney U tests, and percentages in exact Pearson's chi-squared tests.

## Results

**Figure 1:**
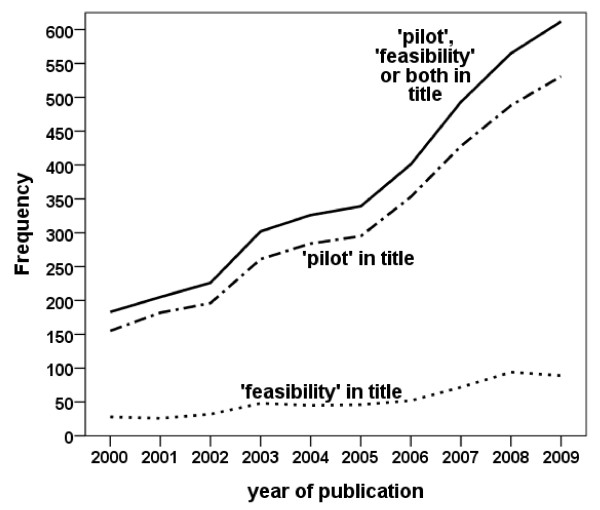
**Frequencies of papers identified with 'pilot', 'feasibility' or both in the title (searched on 21^st ^February 2011)**.

After removal of duplicates our EMBASE/MEDLINE search identified 3,581 papers (see Table 1). In order to achieve a sample of 50 suitable papers a further 25 were rejected for the reasons shown in Table 1. The final two papers were excluded at a late stage because the words 'pilot' or 'feasibility' in the title did not relate to the trial (in one 'pilot' was part of the name of the intervention and in the other the intervention aimed to increase the feasibility of a further procedure). When the search was repeated on 21^st ^February 2011 the number of papers had increased to 3652 (Figure 1). The frequency of papers rose steeply with time. Although not formally evaluated it is likely that, as in the sample of papers selected for review, a third would not meet our eligibility criteria. The majority (3120, 85%) of papers had the word 'pilot' in the title; 479 (13%) had the word 'feasibility'; and 50 (1%) had both.

**Table 1:** Results of the literature search, and exclusions from the selected sample

MEDLINE/EMBASE search		Number of papers
**1**	pilot.ti	40741

**2**	feasibility.ti	16179

**3**	1 or 2	56430

**4**	limit 3 to randomized controlled trial	7553

**5**	limit 4 to english language	7398

**6**	limit 5 to humans	7348

**7**	limit 6 to yr = "2000 - 2009"	5965

**8**	remove duplicates from 7	3581

**RANDOM SAMPLE OF PAPERS**		

Papers randomly selected from 8		75

**EXCLUDED PAPERS (n = 25)**		

Remove cross-over trials (n = 14)		61

Remove trials with only one arm (n = 1)		60

Remove historically controlled trials (n = 2)		58

Remove non-randomized trials (n = 2)		56

Remove letters (n = 1)		55

Remove brief reports (n = 1)		54

Remove study protocols (n = 1)		53

Remove review articles (n = 1)		52

Remove articles where 'pilot' or 'feasibility' in the trial did not relate to the trial (n = 2)		50

Table 2 describes the characteristics of the trials reported in the papers selected for review [10 - 59] The percentages with 'pilot' or 'feasibility' in the title were similar to those amongst the 3,652 papers identified. In four titles [32,41,45,46] the word 'feasibility' described the trial: in the other four [30,38,42,43] it described the intervention. Over half of the trials, (28, 56%, CI 41% to 70%) evaluated drugs. The majority (29, 58%) were single centre trials, 19 (38%) were multi-centre, and in two cases we were unable to determine whether one or more centres were involved. Most trials consisted of one active and one control arm, but the drug trials often had active arms at several doses and 8/28 (29%) had no placebo arm. One non-drug trial was unusual because it had 12 arms. It was a factorial trial carried out over the internet with one factor being six ways of presenting treatment effects to participants crossed with a second factor being the order of eliciting their understanding of treatment consequences from visual analogue or category rating scales [20]. The drug trials were more likely than non-drug trials to incorporate blinding (75% *vs *32%, RR 2.4, CI 1.3 to 4.7), or to be described as double blinded (54% *vs *5%, RR 11.8, CI 2.3 to 167.6). Most papers didn't justify the sample size; 11 presented a power calculation which in all but one related to efficacy (the exception being the internet trial which related to correlation between alternative scales for assessing understanding of treatment consequences [20]); and 3 included non-statistical justifications (one stated the size to be adequate for a pilot study providing the chance to see if there were trends between active and placebo arms [53], the second didn't present a power calculation on the grounds that it was a pilot study but the size of 60 was based on safety data for other indications and was an achievable number [28], while the third was designed as a pilot, no significant differences were anticipated, and the size was chosen based on feasibility for a single-site study [10]). The median achieved sample size was 34 but there were three large trials: the first recruited 425 adolescents to test a sleep educational program in secondary schools [23]; the internet trial [20] recruited 998 people after sending out approximately 700,000 emails; and 3,318 people were recruited from 653,417 information packs mailed in a screening trial for lung cancer [32].

**Table 2:** Characteristics of the drug and non-drug trials

		Drug (n = 28)	Non-drug (n = 22)
**Index word in title**	**pilot**	25 (89%)	17 (77%)
	**feasibility**	1 (4%)	4 (18%)
	**both**	2 (7%)	1 (5%)

**Number of centres**	**single**	18 (64%)	11 (50%)
	**multi-centre**	10 (36%)	9 (41%)
	**unclear/not stated**	0	2 (9%)

**Active arms**	**1**	15 (54%)	16 (73%)
	**2**	8	5
	**3**	4	0
	**4**	1	0
	**12**	0	1

**Control/placebo arm**		20 (71%)	18 (82%)

**Any blinding mentioned**		21 (75%)	7 (32%)

**Stated to be double blinded**		15 (54%)	1 (5%)

**Target sample size**	**not stated**	18 (64%)	15 (68%)
	**stated with no justification**	2 (7%)	1 (5%)
	**non-statistical justification**	3 (11%)	0
	**statistical justification**	5 (18%)	6 (27%)

**Actual sample size**	**median**	34	30.5
	**min-max**	10-87	6-3318
	**sample size not stated**	n = 1	n = 0

**Figure 2:**
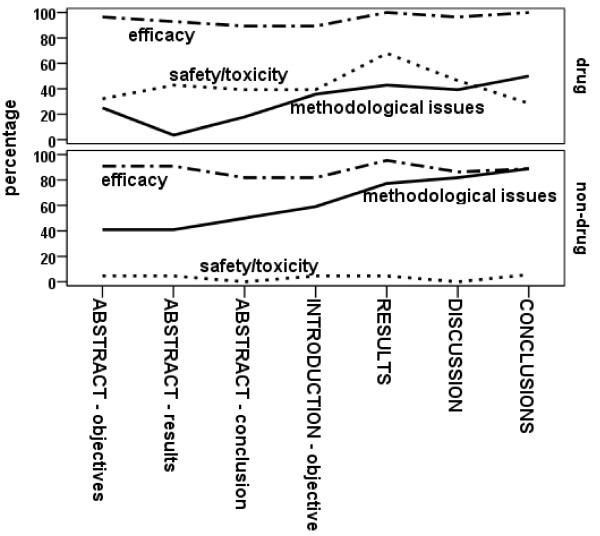
**Percentage of papers in which feasibility, efficacy and safety/toxicity objectives, results, discussion or conclusions were presented (percentages for the Conclusions section based on the 32 papers including one)**.

Figure 2 presents the percentage of papers in which methodological issues, efficacy, and safety/toxicity were explicitly stated as objectives, addressed with numerical results, discussed to an extent rated greater than minimal, or where conclusions were drawn. High percentages relating to efficacy can be seen for both drug and non-drug trials. The drug trials also addressed safety/toxicity issues, whereas the non-drug trials were more likely to additionally address methodological issues. In Table 3 the specific methodological issues are detailed. Recruitment and retention were frequently, though not always addressed to some extent: most papers with tabular/graphical presentation covered the issues in a CONSORT flowchart. Compliance/adherence to intervention was included in a few CONSORT flowcharts, but was more frequently addressed in text. Other aspects of intervention examined included cost and duration. The one paper that presented tabulated/graphical results relating to outcome assessment portrayed values elicited with category rating scales mapped onto a visual analogue scale format [20]. We rated the average costs per randomization presented in one paper [49] as detailed results relating to randomization procedures, the two papers with brief results on this topic commented on a failure in the randomization service [12] and gave numbers and reasons why potential participants missed being randomized [38]. Although our sample included 19 multi-centre trials only one presented numerical results evaluating procedures involved (ratings of cooperation between different centres [49]).

**Table 3:** Methodological issues that were addressed numerically with frequencies of participants or other statistical methods

Issue		Drug (n = 28)	Non-drug (n = 22)
**Recruitment**	**none**	21 (75%)	9 (41%)
	**brief coverage**	5 (18%)	4 (18%)
	**detailed coverage**	1 (4%)	3 (14%)
	**tabulated/figure**	1 (4%)	6 (27%)

**Retention**	**none**	16 (57%)	9 (41%)
	**brief coverage**	6 (21%)	7 (32%)
	**detailed coverage**	0	0
	**tabulated/figure**	6 (21%)	6 (27%)

**Compliance/adherence with the intervention**	**none**	14 (50%)	10 (46%)
	**brief coverage**	7 (25%)	7 (32%)
	**detailed coverage**	5 (18%)	1 (5%)
	**tabulated/figure**	2 (7%)	4 (18%)

**Blinding procedures**	**brief coverage**	2 (7%)	1 (5%)

**Acceptability of the intervention to participants**	**brief coverage**	1 (4%)	1 (5%)
	**detailed coverage**	0	1 (5%)
	**tabulated/figure**	0	2(9%)

**Other aspects of the intervention**	**brief coverage**	1 (4%)	2 (9%)
	**detailed coverage**	0	1 (5%)
	**tabulated/figure**	0	2 (9%)

**Outcome assessment**	**brief coverage**	1 (4%)	4 (18%)
	**detailed coverage**	1 (4%)	0
	**tabulated/figure**	0	1 (5%)

**Randomization procedure**	**brief coverage**	0	2 (9%)
	**detailed coverage**	0	1 (5%)

**Acceptability of trial procedures**	**brief coverage**	1 (4%)	0
	**detailed coverage**	0	3 (14%)

**Logistics of multi-centre procedures**	**detailed coverage**	0	1 (4%)

The emphasis on efficacy demonstrated in Figure 2 is explored in Table 4. Statistically significant differences between groups were reported in 19 (38%) of papers, but we were unable to determine whether these related to pre-stated primary outcomes or were opportunistic reporting. Where significant differences between groups were not reported sometimes significant within group differences were. In 43 (86%) the Discussion section included a statement that the trial was too small: a similar statement was made in 24 (48%) of the Abstracts. Efficacy was addressed in the Discussion by all authors and in 26 (52%) it was the major focus. The take home message on efficacy from the Abstract section was rated to be that the intervention was beneficial in 24 (48%), and showed promise in 13 (26%). In 8 (16%, CI 7% to 28%) of the Abstracts there was no mention of efficacy.

**Table 4:** Results, discussion and conclusions concerning efficacy

		Drug (n = 28)	Non-drug (n = 22)
**ABSTRACT**			

**Take home message**	**no mention of efficacy**	4 (14%)	4 (18%)
	**not shown to have benefit/no benefit**	4 (14%)	1 (5%)
	**intervention shows promise**	8 (29%)	5 (23%)
	**intervention beneficial**	12 (43%)	12 (55%)

**Mentioned that further/larger trials needed**		13 (46%)	11 (50%)

**RESULTS**			

**Statistically significant**	**none**	12 (43%)	9 (43%)
**results relating to efficacy**	**within groups only**	5 (18%)	4 (19%)
	**between groups**	11 (39%)	8 (38%)^1^

**DISCUSSION**			

**Extent of discussion about**	**minimal**	1 (4%)	3 (14%)
**efficacy**	**substantial**	11 (39%)	9 (41%)
	**major focus of section**	16 (57%)	10 (46%)

**Mentioned that the sample size was too small**		24 (86%)	19 (88%)

1 - One paper reporting a non-drug trial didn't report any significance tests or confidence intervals and was excluded from these figures

As shown in Table 5, while most authors (39, 78%, CI 64% to 88%) did mention methodological issues in the Discussion it was often to a minimal extent. We rated the discussion to be substantial or the major focus of the section for 28 (56%, CI 41% to 70%), and covered to greater depth in the papers reporting non-drug compared to drug trials (P = 0.002). Most papers mentioned future trials but it was usually to an extent we rated as minimal: as with the coverage of feasibility issues more generally the depth of discussion concerning future trials was rated to be greater in the paper reporting non-drug trials (P = 0.002). Papers reporting non-drug trials were also more likely to include a methodological conclusion in the Conclusions section if there was one (P = 0.022), and in the Abstract (P = 0.031). The groups were similar with respect to whether the authors were actually conducting a subsequent trial with only 6 (12%, CI 5% to 23%) overall stating that one was underway or scheduled to start in the near future.

**Table 5:** Discussion and conclusions about planning further studies

		Drug (n = 28)	Non-drug (n = 22)	P
**Coverage of feasibility issues in the Discussion section**	**none**	8 (29%)	3 (14%)	0.002^1^
	**minimal**	9 (32%)	2 (9%)	
	**substantial**	9 (32%)	7 (32%)	
	**major focus of section**	2 (7%)	10 (46%)	

**Extent of discussion or recommendations about planning future trials**	**none**	6 (21%)	1 (5%)	0.002^1^
	**minimal**	21 (75%)	13 (59%)	
	**substantial**	1 (5%)	8 (36%)	
	**major focus of section**	0	0	

**One or more conclusion about** **methodological issues in a conclusions section**		7/14 (50%)	16/18 (89%)	0.022^3^

**One or more conclusion about** **methodological issues in the Abstract**		5 (18%)	11 (50%)	0.031^3^

**Conducting a subsequent trial** **or about to start one in the near future**		3 (11%)	3 (14%)	1.000^3^

1 - Mann Whitney U test

2 - Restricted to papers with a Conclusions section

3 - Exact Pearson's chi-squared test

## Discussion

For the main part the pilot and feasibility trials in our review did not primarily address methodological issues. Although lessons learnt about planning trials were discussed in the majority of papers it was often to an extent we rated as minimal, and in only 6 (12%) of papers was it stated that the authors were actually conducting a subsequent trial or about to start one. This frequency is not dissimilar to the 9% of pilot studies reviewed by Lancaster et al [2], subsequently found to have been followed by a larger study [4]. Even though authors themselves may not proceed to a larger RCT it is possible that others reading the paper will. Our impression was that many of the trials fell into the latter of Loscalzo's [9] two classes: namely those designated as pilots *a posteriori *possibly after failing to demonstrate the hoped for effects or because of inadequate sample size. In all but one of the 11 papers including a power calculation, sample size was determined to achieve power in testing efficacy. In other papers it was impossible to be sure what *a priori *objectives were from the published paper alone, but since methodological issues were discussed to greater depth in the non-drug trials, they are more likely to fall into Loscalzo's class of *a priori *pilot trials. Even amongst the non-drug trials there was generally an emphasis on efficacy.

We interpreted efficacy as the examination of change in an outcome variable not clearly related to safety/toxicity. The final group of scientific objectives for pilot studies listed by Thabane et al [5] includes obtaining estimates of the treatment effect and its variance. Thabane et al also discuss the distinction between pilot and proof-of-concept studies which they define as a clinical trial carried out to determine if a treatment (drug) is biologically active or inactive. Arnold et al [3] include the assessment of mechanisms, possibly using surrogate measures, as a legitimate objective of pilot trials, to establish proof-of-principal and potential efficacy. Many of the papers in our review may lie on the margins between pilot and proof-of-concept investigations and their emphasis on efficacy should perhaps be interpreted in this light, however none were described as proof-of- either concept or principal studies in their title [10 - 59], though one [42] was described as a phase II pilot study.

We specifically selected pilot/feasibility trials that incorporated random allocation. Many methodological issues do not need to be examined in the context of an RCT: for example larger numbers would be available from routinely collected data; it is generally easier to conduct a single group study; and greater depth of understanding of the acceptability of interventions is obtained from qualitative research. Some issues that cannot be satisfactorily investigated other than in the context of a randomized trial are the percentage consenting to randomization, retention in intervention and control groups, whether blinding can be maintained, and whether all components of the protocol work together. Given the burden of research governance concerning RCTs, it would seem sensible to evaluate specific aspects of a protocol using simpler studies wherever possible. In Table 6 we indicate with a tick methodological issues that require piloting in the context of an RCT, those marked with a cross could be assessed in other types of feasibility study.

**Table 6:** Methodological issues that need evaluation in the context of an RCT

Issue	Needs to be evaluated in the context of a randomized pilot trial	Comments
**Sample size calculation**	✗	The numbers in a pilot RCT are unlikely to be adequate to get accurate estimates of effect size of variances.

**Eligibility**	✗	

**Recruitment**	✓	Referrals from clinicians are likely to depend on the RCT context.

**Consent**	✓	Consent rates in the RCT context are unlikely to be accurately estimated from asking about likely consent beforehand

**Randomization procedures**	✓	

**Blinding procedures**	✓	

**Compliance/adherence to intervention**	✗	Though, this could potentially depend on preference amongst interventions offered in the main trial

**Acceptability of intervention**	✗	Though, this could potentially depend on preference amongst interventions offered in the main trial

**Cost and duration of intervention**	✗	

**Outcome assessment**	✗	

**Selection of most appropriate outcomes**	✗	

**Retention**	✓	Retention may differ between experimental and control groups, and may depend on treatment preferences

**Logistics of multi-centre trial**	✓	

**All components of the protocol work together**	✓	

Thabane et al [5] recommend that explicit criteria indicating that a subsequent trial is feasible should be stated: they describe the criteria set out in advance for proceeding from the pilot to the main Prophylaxis of Thromboembolism in Critical Care Trial (PROTECT) [62], also reviewed by Arnold et al [3]. In contrast, Gardener et al [63] describe a case study where unanticipated problems arising during a pilot lead to abandoning a subsequent RCT even though pre-stated objectives indicated the methodology to be feasible. They identify the availability of funding and the contemporary health service environment as issues likely to impact on the decision to proceed. We believe that studies evaluating the feasibility of trial procedures are essentially exploratory in nature. Researchers should examine carefully the success of procedures and react to unanticipated problems to get the best possible design for their next trial. Aspects of the design aren't decided in isolation, predicted recruitment under a set of eligibility criteria may be adequate if there is a change in outcome variable for example. If extensive changes are made it may be advisable to retest the feasibility of the protocol.

Others have searched for pilot/feasibility trials adopting different criteria leading to different populations of papers surveyed. Lancaster et al [2] could find no guidance on how to search MEDLINE for pilot/feasibility trials, and restricted their search to papers in six top ranking medical journals with the words 'pilot' or 'feasibility' in the title, abstract or keywords: of the 115 hits retrieved 25 (22%) were not suitable for a variety of reasons. Arain et al [4] repeated Lancaster et al's search procedure seven years later with a rate of unsuitable papers (30%) similar to ours of 33%. Arnold et al [3] initially searched MEDLINE for pilot trials in critical care medicine, but then canvassed known clinical investigators because of the poor indexing of pilot trials. They describe five pilot trials fulfilling the requirements of either addressing methodological issues relating to the feasibility of subsequent trials, or assessing mechanisms of intervention. Reviews based on published papers do not address internal pilots which continue into the main phase and are unlikely to be reported separately, though where a decision is taken not to continue the pilot phase could be written up. We chose to include only full papers in our study: different issues may arise amongst pilot or feasibility trials that are published as letters or brief reports. Sampling from all journals covered by MEDLINE and EMBASE and restricted to trials incorporating random allocation, the characteristics of the pilot/feasibility trials we found is not unexpected. The majority bear little resemblance to the recent definitions proposed for pilot/feasibility studies.

## Conclusions

Our main findings are that RCTs described by their authors as pilots or addressing feasibility most commonly focus on efficacy, in just over a half (56%) were issues that might inform the planning of a subsequent trial addressed in reasonable depth. In addition to efficacy pilot drug trials also addressed safety, while pilot non-drug trials were more likely to additionally address methodological issues. While the median sample size was quite small at 34, there were three trials recruiting over 100 participants, demonstrating that sample size very much depends on circumstances even in the context of pilot/feasibility trials.

## Competing interests

The authors declare that they have no competing interests.

## Authors' contributions

MS carried out the literature search, created the review form and reviewed the papers under the supervision of RMP and MW. RMP and MW conceived the study, supervised its conduct and reviewed some of the papers. RMP drafted an initial version of the manuscript: all authors contributed to and approved the final manuscript.

## Pre-publication history

The pre-publication history for this paper can be accessed here:

http://www.biomedcentral.com/1471-2288/11/117/prepub
